# *β‐Catenin*‐Deficient Intervertebral Disc Cells Reduce Chemotactic Transcription and Myeloid Cell Recruitment to Injured Discs of Mice

**DOI:** 10.1111/acel.70740

**Published:** 2026-10-01

**Authors:** Tori M. Kroon, Jazz Munitz, Anna Ranzenigo, Martin Umali, William Wang, Jonathan J. Huang, Abraham J. P. Teunissen, Nilsson Holguin

**Affiliations:** ^1^ Department of Orthopedics Icahn School of Medicine at Mount Sinai New York New York USA; ^2^ Biomedical Engineering and Imaging Institute Icahn School of Medicine at Mount Sinai New York New York USA; ^3^ Icahn School of Medicine at Mount Sinai Cardiovascular Research Institute New York New York USA; ^4^ Icahn School of Medicine at Mount Sinai Icahn Genomics Institute New York New York USA

**Keywords:** aging, genetic mouse model, inflammation, injury, Wnt signaling

## Abstract

Aging exacerbates the incidence of discogenic chronic low back pain by excessive inflammation and immune cell presence. Aging reduces intervertebral disc (IVD) Wnt signaling, which is associated with IVD cell differentiation, IVD cell proliferation and anti‐inflammatory signaling. However, it is unclear how in vivo Wnt signaling‐absent IVD cells, which model aged IVD cells, regulate chemotactic signaling and immune cell recruitment. We hypothesized that Wnt signaling‐absent IVD cells attenuate chemotactic transcription and immune cell recruitment to naive IVD and in response to IVD injury. First, because aging reduces Wnt signaling in all IVD cells, we deleted *β‐catenin* using the AcanCreERT2. *β‐catenin* deletion reduced monocyte chemoattractant protein‐1 (MCP1/CCL2) *Ccl2* in male and female IVDs by 58% and 79%, respectively (*n* = 5–6/sex). Using positron emission tomography/computed tomography (PET/CT) imaging of myeloid cells in 5‐month‐old mice (*n* = 4 IVD/sex/grp), deletion of *β‐catenin* reduced myeloid‐derived cell burden in naive IVD by 79% and attenuated IVD injury‐induced recruitment by 48%. Next, because nucleus pulposus (NP) cells begin losing Wnt signaling early in life, we deleted *β‐catenin* using the ShhCreERT2. Compared to RNA‐sequencing of WT IVD post injury (*n* = 3/grp), deletion of *β‐catenin* in NP cells impaired the activation of immune cell pathways, such as MCP1/CCL2. Together, the chemotactic transcriptomic response and myeloid cell accumulation due to IVD injury were impaired by the absence of IVD Wnt signaling transcription cofactor *β‐catenin*. These data suggest that low‐grade IVD inflammation and chronic IVD immune cell accumulation in aged IVD cells are regulated by IVD Wnt signaling.

## Graphical abstract

(Left) In young discs, Wnt signaling‐replete nucleus pulposus cells secrete ECM components and, upon disc injury, cytokines to recruit myeloid cells. (Right) Whereas, *β‐catenin*‐deficient disc cells, modeling age‐related absence of Wnt signaling, exhibit reduced anabolic activity and, upon disc injury, secrete fewer pro‐inflammatory cytokines and recruit fewer myeloid cells.

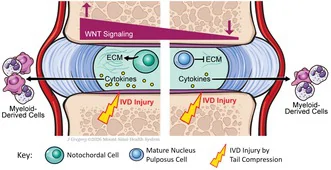

## 1 Introduction

Aging promotes intervertebral disc (IVD) degeneration (IVDD), which is a leading cause of chronic low back pain (cLBP) (Sima and Diwan 2025; Patil et al. 2018). Age‐related cLBP is associated with impaired IVD healing from chronic inflammation, which promotes nerve ingrowth and immune cell recruitment (Dou et al. 2025), but the mechanisms by which aging exacerbates the development of discogenic cLBP are unclear. IVDD is characterized by a narrowing of IVD height due to mechanical weakening resulting from extracellular matrix (ECM) breakdown and IVD dehydration. However, aging does not necessarily lead to IVDD (Adams and Roughley 2006), which can be asymptomatic of pain (Boden et al. 1990; Fujii et al. 2019), and cLBP can occur without IVDD (Sima and Diwan 2025). To address these disparities, recent data suggest that impaired neutrophil recruitment and resolution by anti‐inflammatory NSAID administration are associated with cLBP (Parisien et al. 2022). Therefore, clarifying the role of aged IVD cells in immune cell recruitment during inflammation could offer further insight into the development of cLBP.

The IVD has low cellular density (Boubriak et al. 2013) with low ECM turnover (Liang et al. 2022), low vascularization, and is commonly referred to as an ‘immuno‐privileged’ tissue, though resident immune cells do reside in the IVD (Y. Wang et al. 2025). Cellularly, aged and degenerated IVD incur a cellular switch of resident cells from high ECM‐producing, immature NP cells to lower ECM‐producing, mature NP cells (Liang et al. 2022) that are also triggered by notochordal progenitor NP cells to stimulate glycosaminoglycan and aggrecan core protein synthesis. Therefore, the current paradigm is that early signs of IVDD begin with this cellular switch of NP cells, but there is not a potential pathophysiological rationale for this IVD cellular switch.

Wnt signaling is a molecular pathway that can regulate cell differentiation, cell proliferation, and activity (Holguin and Silva 2018). Transcription cofactor *β‐catenin* is regulated by canonical Wnt signaling, where activation of this pathway leads to translocation of *β‐catenin* from the cytosol to the cell nucleus and binding to T‐cell factor and the lymphoid enhancer factor (TCF and LEF) co‐transcriptional factors to activate downstream Wnt signaling target genes (Liu et al. 2022). Transcriptional factors for notochordal NP cells require TCF/LEF signaling, and notochordal cell fate is driven by Wnt signaling (Winkler et al. 2014). Aging and IVD injury both promote maturation of IVD cells (Holguin and Silva 2018; Kondo et al. 2011) as evidenced by loss of Wnt signaling in the IVD and accumulation of chondrogenic‐expressing cells (Wu et al. 2021).

The role of Wnt signaling‐deficient aged IVD cells in IVD injury remains unclear because the distinction among their roles in IVD cell differentiation, IVD cell proliferation, and IVD cell inflammatory signaling has not been clarified. Deletion of Wnt signaling transcription factor *β‐catenin* confirms the role of Wnt signaling on IVD mechanical function, which is associated with overall IVD health. Deletion of *β‐catenin* reduces IVD compressive stiffness and stabilization of *β‐catenin* increases IVD compressive stiffness (Holguin and Silva 2018). Decreased IVD compressive stiffness indicates weakened load‐bearing capacity that would allow more IVD deformation, while an increased IVD compressive stiffness would allow the IVD to resist deformation and withstand more load. Severe IVDD is functionally characterized by stiffening from fibrosus, but early IVDD without fibrosus, such as occurs with aging, begins with reduced stiffness. Cellularly, Wnt signaling is a molecular pathway that drives notochordal cell fate (Winkler et al. 2014) and proliferation by activating *cyclin‐d, c‐myc*, and other genes (Wu et al. 2021). However, aging and IVD injury both promote IVD cell maturation (Holguin and Silva 2018; Kondo et al. 2011) by loss of IVD cell Wnt signaling (Wu et al. 2021). Lastly, Wnt signaling in IVD cells may also play an important role in regulating inflammatory chemokines and cytokines. In vitro, Wnt signaling forms a positive feedback loop with TNF‐α, where suppression of Wnt signaling reduces TNF‐α expression in NP cells (Hiyama et al. 2013).

Here, we set out to identify the chemotactic transcriptomic signals and track the systemic recruitment of myeloid‐derived cells from IVD injury in an in vivo model of IVD cell‐specific absence of Wnt signaling as occurs with aging, while minimizing the aging‐related changes exogenous to the IVD (Sadowska et al. 2018) to help clarify the IVD cell‐specific contribution to age‐related IVD healing. We used three mouse models: (1) two genetic mouse models to delete Wnt signaling transcription factor β*‐catenin* that model IVD aging and (2) an overloading injury model of IVDD to control the timing and degree of IVDD.

## 2 Methods

### 2.1 Mice

To suppress Wnt/*β‐catenin* signaling in the whole IVD at young‐adulthood and avoid disruption of spinal developmental (Kondo et al. 2011), tamoxifen‐inducible Acan‐CreERT2 mice (B6.Cg‐Acan^tm2(cre/ERT2)Crm^/J, JAX: stock # 019148) were bred with *β‐Catenin* ^fl/fl^ mice (B6.129‐Ctnnb1^tm2Kem^/KnwJ, Jax: stock # 004152) and GFP mice ([Tg(TCF/Lef1‐HIST1H2BB/EGFP)61Hadj/J], Jax: stock # 013752). This will target *β‐catenin* deletion in aggrecan‐expressing cells of the spine, such as NP cells, annulus fibrosus (AF) cells, and the vertebral growth plate and endplate (Burt et al. 2024). Male and female AcanCreERT2/*β‐Catenin* ^fl/fl^ (conditional knockout, cKO^ACN^, *n* = 6 per sex) and *β‐Catenin* ^fl/fl^ littermate mice (wild‐type, WT, *n* = 6 per sex) were housed in a 12‐h light/dark cycle and fed standard chow up to 4 months of age. Next, to suppress Wnt/*β‐catenin* signaling in the NP of the IVD at young‐adulthood (Silva and Holguin 2020) and avoid disruption of spinal development (Kondo et al. 2011), tamoxifen‐inducible Shh‐CreERT2 mice (B6.129S6‐Shh^tm2(cre/ERT2)Cjt^/J, JAX: stock # 005623) were bred with *β‐Catenin* ^fl/fl^ mice (B6.129‐Ctnnb1^tm2Kem^/KnwJ, Jax: stock # 004152). Female ShhCreERT2/*β‐Catenin* ^fl/fl^ (conditional knockout, cKO^SHH^, *n* = 3) and *β‐Catenin* ^fl/fl^ littermate mice (wild‐type, WT, *n* = 3) were maintained under ideal conditions, housed independent of genotype in a 12‐h light/dark cycle and fed standard chow up 4 months of age. At 4 months‐of‐age for genetic KO models, the chow for all mice was changed to tamoxifen chow (Inotive, cat no.: TD.130859) for 4 days to induce genetic recombination and delete *β‐Catenin* in the respective mouse models, followed by 1 month of regular chow to allow for potential structural changes or tail compression (TC). Lastly, to determine the influence of advanced age on injury‐induced upregulation of cytokines and chemokines, 5‐, 12‐ and 24‐month‐old, male and female mice were tail compressed for 24 h. Mice were euthanized by CO_2_ administration and coccygeal caudal IVD were harvested for qpcr. All experiments were performed under IACUC approval, no animals were excluded, and data analyzed in a blinded fashion.

### 2.2 RNA Extraction and qPCR

IVDs CC10‐13 were harvested, flash frozen in liquid nitrogen, pulverized using the Mickrodismembrator (B. Braun Biotech International Mikro‐Dismembrator S, Germany), and suspended in TRIZOL (Ambion, Austin, TX, USA). RNA isolation and purification steps were followed (RNeasy Mini Kit, Qaigen, Valencia, CA, USA), including on‐column DNase treatment to remove contaminating genomic DNA, and RNA was quantified (Nanodrop, Thermo Fisher Scientific, Waltham, MA, USA). CDNA was synthesized (iScript, Bio‐Rad, Hercules, CA, USA) from 400 ng of total RNA. To confirm deletion, custom sybr green primers were made for *β‐catenin* (Integrated DNA Technologies, Coralville, IA, USA, Forward—AAA GTT GTT TGT ACA GAG TGT GGA, Reverse—AAG ACA CAA ATC TTT GAG CCT GTG) and normalized to *gapdh* (Forward—ACC ACA GTC CAT GCC ATC AC, Reverse—TCC ACC ACC CTG TTG CTG TA). For other genes, the following Taqman probes were used (Life Technologies, Carlsbad, CA, USA); *dkk1* (Mm00438422_m1), *lef1* (Mm00550265_m1), *ccnd1* (Mm00432359_m1), *ccl2* (Mm00441242_m1), *il1b* (Mm00434228_m1), *S100a9* (Mm00656925_m1), *serpina1a* (Mm02748447_g1), *il6* (Mm00446190_m1), *tagln* (Mm00441661_g1), *brachyury* (Mm00436877_m1), *krt8* (Mm04209403_g1), *cd109* (Mm00462151_m1), *scx* (Mm01205675_m1), *aggrecan* (Mm00565794_m1), *collagen1* (Mm00801666_g1), *mmp3* (Mm00440295_m1), *collagen2* (Mm01309565_m1), and *adamts5* (Mm00478620_m1). Relative gene expression was normalized to *gapdh* (Mm99999915_g1) for each group and then experimental values were normalized to the average of the Male WT values ($2^{-\Delta \Delta C_{T}}$).

### 2.3 Tail Compression

Mechanical injury of the IVD by TC is an in vivo rodent model of IVDD, whose advantages include control of IVDD severity, timing and spinal level of IVDD, and the ease and reproducibility of implementation (Stinnett‐Donnelly et al. 2007). Briefly, CC7 and CC9 were identified with preoperative radiographs by in vivo X‐ray scanning (Faxitron). While the mice were under anesthesia with isoflurane (2.5% vol), two 23‐G needles serving as pins were transcutaneously implanted through CC7 and CC9, and postoperative radiographs were used to confirm their proper placement (Figure S1). Subsequently, compression rings (Cat #90107A029, McMaster, Robbinsville, NJ, USA) were attached externally to the needles to apply a compressive mechanical load via tightening of four screws with compressive springs (Cat #91858A203, Cat #9001T24, McMaster). Following injection of buprenorphine (1 mg/kg s.c.) for pain relief, 2.25 N of load was applied for 1 week to induce IVDD to CC7‐8 and CC8‐9. The estimated stress applied to the IVD based on mouse IVD geometry is 1 MPa, which is roughly equivalent to the change in intradiscal pressure from standing up from a chair (Wilke et al. 1999). IVD CC5‐6 and CC10‐11 were not compressed and served as internal controls.

### 2.4 Cryosectioning and Immunohistochemistry

To assess Wnt signaling activity using the GFP reporter mouse, lumbar motion segments (L1–3) were fixed in zinc formalin for 48 h, decalcified in ethylenediaminetetraacetic acid (EDTA) for 2 weeks, submerged in 5% sucrose for 1 h then 30% sucrose overnight. Sections were embedded in optimal cutting temperature (OCT), cryosectioned at 12 μm thick, then mounted using Invitrogen ProLong Gold Antifade Mountant with DNA Stain DAPI (P36931, ThermoFisher Scientific, MA, USA) and cover slipped. Motion segments were imaged on the Zeiss Imager Z1 under gfp and dapi lighting and active wnt signaling was denoted by green fluorescence near the cell nuclei. NP images and quantification were not included due to histological artifacts while processing.

IVD for immunohistochemical staining were run in a single batch. Control segments (CC5‐6 and CC10–11) and TC segments (CC7‐9) (Figure S2) were fixed in zinc formalin for 48 h, decalcified in ethylenediaminetetraacetic acid (EDTA) for 2–3 weeks, submerged in 70% ethanol, embedded in paraffin, then sectioned (8 μm). For quantification of cd11b (ab133357, 1:1000, Abcam, Cambridge, UK) counterstained with Light Green solution. Outer AF and peripheral tissue (excluding IVD fat pad, muscle, and connective tissue) were quantified as the ROI and color thresholded for teal and brown (cd11b positive). Values graphed are the brown area divided by the total area (teal + brown) representing the percentage of the ROI stained positive for cd11b. Samples with histological anomalies, such as folding or tears, were excluded from analyses.

### 2.5 Bulk RNA Sequencing and Analysis

From each WT and cKO^SHH^ mouse, IVDs CC7‐9 were pooled to serve as one ‘compressed’ sample (TC) and IVDs CC5‐6 and CC10‐11 were pooled to serve as 1 ‘control’ sample (Con) (Figure S1) (RIN 6.4 ± 0.8, average of 26.7 million high quality reads per sample). RNA‐Sequencing was run (Illumina HiSeq 4000) using the KAPA mRNA Hyperprep Kit (KK8581) library protocol. Bulk RNA sequencing data were analyzed using R software and packages. Raw count data were normalized using trimmed mean of M‐values (TMM) method used in “edgeR” package (v4.0.16). Differentially expressed genes (DEGs) between samples were identified with the “edgeR” package, with default package quality control and threshold criteria of FDR > 0.1 and |log_2_FC| > 1, and the summed gene count > 30. FDR correction was applied using the Benjamin–Hochberg procedure. Principal component analysis (PCA) plots were generated after analysis and “vst” selection by the “DESeq2” package (v1.42.1) and with graphical visualization using the “ggplot2” package (v3.5.1). Gene names were standardized using the “org.Mm.eg.db” package (v3.18.0) and Gene ontology (GO) was performed with the “GOexpress” package (v1.36.0) in the ‘BP’, “Biological Process” setting. A list of the top 20 most significantly up‐ and down‐regulated pathways was obtained for the genotype comparison (Controls: WT vs. cKO^SHH^) and in response to IVD injury (TC) for both the WT (WT: Control vs. TC) and cKO^SHH^ groups (cKO^SHH^: Control vs. TC). Each pathway containing a list of genes was then analyzed in the Reactome Pathway Browser to determine its categorical biological process. Heatmaps of significant pathways were generated with the “pheatmap” package (v1.0.12). Volcano plots were created using the “tibble” package (v3.2.1) and the “ggplot2” package (v3.5.1).

### 2.6 Formulating PERFECTA‐HDL

1,3‐bis[[1,1,1,3,3,3‐hexafluoro‐2‐(trifluoromethyl)propan‐2‐yl]oxy]‐2,2‐bis[[1,1,1,3,3,3‐hexafluoro‐2‐(trifluoromethyl)propan‐2‐yl]oxymethyl]propane (PERFECTA) was synthesized according to (Tirotta et al. 2014) and formulated into PERFECTA‐HDL as previously described (Maier et al. 2023).

### 2.7 Radiolabeling PERFECTA‐HDL

A PBS suspension of PERFECTA‐HDL (0.24 mL with a 1 mg/mL apoA1 concentration, 1.0 eq) was diluted with 1 M carbonate buffer until a pH of 8.5 was reached. A solution of DFO‐*p*‐NCS in DMSO (19.25 μg, 3.0 eq, 7 μL) was added. The mixture was incubated at 37°C for 3 h and manually shaken every 15 min. The suspension was then transferred to a centrifugal filtration tube (Vivaspin, 1 MDa MWCO), diluted with PBS (5 mL) and spun at 4000 g at 4°C until 1 mL remained. PBS (5 mL) was added and the sample was again concentrated to 1 mL. This was repeated twice. The DFO‐labeled PERFECTA‐HDL was stored in the dark at 4°C and used for radiolabeling within 7 days. A solution of ^89^Zr in oxalic acid (6.0 μL, 1,19 mCi) was diluted with PBS (0.10 mL) and neutralized using an aqueous Na_2_CO_3_ solution (1 M, approx. 4 μL) to reach a pH between 6.8–7.4 (checked using pH paper). DFO‐functionalized PERFECTA‐HDL (1.0 mL) was added and the reaction incubated for 30 min at 37°C while shaking at 300 rpm using a thermomixer. Completion of the chelation reaction was verified by radio‐TLC (Lablogic Scan‐RAM, using Agilent iTLC‐SG paper) using an aqueous EDTA solution (50 mM) as eluent. The nanoparticles were allowed to cool to room temperature and transferred to a centrifugal filtration tube (Vivaspin, 1 MDa MWCO), diluted with PBS (5 mL), and spun at 4000 g at 4°C until 1 mL remained. PBS (5 mL) was added and the sample was concentrated to 1 mL. This was repeated twice. An aliquot of the solution was analyzed by radio‐TLC as described above, typically showing a radiochemical purity of > 99%. Radiochemical yields were ~80%.

### 2.8 In Vivo PET/CT Imaging of ^89^Zr‐Labeled PERFECTA‐HDL

3‐month‐old male and female, cKO^ACN^ and WT (*n* = 2 mice for *n* = 4 IVD/group/sex) were fed tamoxifen chow for 4 days to induce genetic recombination and deletion of *β‐catenin*. Mice received a retro‐orbital injection of ^89^Zr‐labeled PERFECTA‐HDL at a dose of 100 ± 10 μCi and the nanoparticles were allowed to circulate for 24 h prior to PET/CT imaging. Immediately before undergoing the scan, the animals were anesthetized using an isoflurane (Baxter Healthcare)/oxygen mix (2% isoflurane for initial induction and 1% for sustained maintenance). Imaging was performed using a Mediso nanoScan PET/CT scanner. A whole‐body CT scan was performed (energy 50 kVp, current 180 μA, isotropic voxel size 0.25mm^3^), followed by a baseline PET scan with an acquisition time of 1 h and 30 min. Coincidences were filtered with an energy window 400–600 keV. The voxel size was isotropic, 0.6mm^3^ in width, and the reconstruction was applied for two full iterations, six subsets per iteration. Mice were allowed to sit for 3 weeks to allow radioactive decay. Following, all mice were subjected to 1 week of TC followed by a secondary RO injection and a 24 h wait time before a secondary (post injury) PET/CT scan. PET data were reconstructed using CT‐based attenuation correction. Reconstruction was performed using the TeraToma 3D reconstruction algorithm from the Mediso Nucline software.

### 2.9 PET/CT Data Analyses

Image analysis was performed using Osirix MD, version 11.0. Reconstructed PET and CT scans were viewed in a 2D transverse orientation, and ROIs were drawn for analysis. For ROIs of visceral organs, ROIs were drawn using the pencil tool in multiple transverse slices to encompass the organ in its entirety. For vertebral ROIs, ROIs were drawn along all transverse slices to encompass the cranial and caudal halves of adjacent vertebral bodies with respect to the IVD of interest. Separate ROIs were also drawn around the IVD specifically (Figure S3). These regions included the two compressed IVD and vertebrae, one cranial control (vertebral body and IVD adjacent to the superior compressed IVD), and one caudal control (vertebral body and IVD adjacent to the inferior compressed IVD). Lastly, PET data were quantified for standard uptake values (SUV), or tracer uptake. This is defined by [pixel value (Bq/mL) × weight of the mouse (kg)/dose (Bq)] × 1000 g/kg. Mean SUV for all ROIs was exported for further quantification using Graphpad Prism (Version 10.5.0).

### 2.10 Statistics

For qpcr quantification, unpaired Student's *t*‐tests were used to compare expression between WT and cKO^ACN^ of male and female mice, with a *p*‐value < 0.05 being considered significant. For pathway analysis, pathways and genes with a false discovery rate (FDR) < 0.1 were considered statistically significant. Bar graphs with standard deviations were used, with raw data points shown for each sample.

The following statistical analysis was performed using Graphpad Prism (Version 10.5.0). For PET analysis, paired *t*‐tests were used to compare the mean baseline and post‐TC SUV values between WT and cKO^ACN^ mice. Separately, unpaired *t*‐tests were used to compare baseline WT vs. cKO^ACN^ mean SUV and post TC WT vs. cKO^ACN^ mean SUV, where a *p*‐value < 0.05 was considered significant. PET/CT data are represented by box plots. The top and bottom lines represented the first and third quartiles, and the center line represented the mean. The top and bottom points represented the maximum and minimum of the data. Cross‐sectional data were compared by unpaired Student's *t*‐tests and longitudinal data were compared by paired Student's *t*‐test. Shapiro–Wilk test was used to test for normality and non‐normal data were compared by Mann Whitney tests. Each point on the graph represented one IVD sample and a *p*‐value < 0.05 was considered significant.

## 3 Results

### 3.1 Deletion of *β‐Catenin* in Aggrecan‐Lineage Cells Reduced *ccl2* Gene Expression and Did Not Regulate Notochordal Cell nor ECM Gene Expression in Female and Male IVD

Aging reduces Wnt signaling in NP cells and AF cells (Silva and Holguin 2020). Therefore, to determine the effect of reduced Wnt signaling in the entire disc, we deleted *β‐catenin* using an aggrecan‐targeting cre driver and evaluated the GFP expression of Wnt signaling using the TCF/Lef:H2B‐GFP Wnt reporter. Compared to WT controls, deletion of *β‐catenin* reduced the number of GFP‐positive AF cells in male IVD by 62%, while female IVD cells were nonsignificantly reduced by 43% (Figure 1A,B). These data indicate that deletion of *β‐catenin* reduced Wnt/*β‐catenin* signaling in the IVD.

**FIGURE 1:**
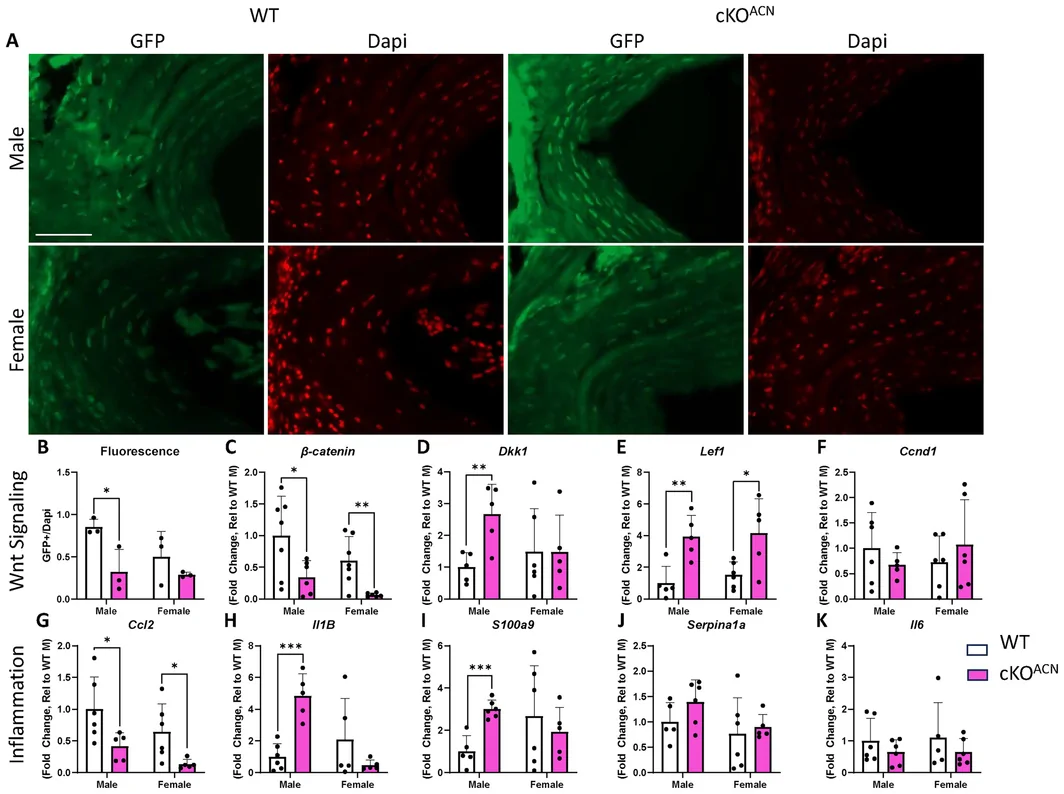
(A) 40× Images of GFP expression from the TCF/Lef:H2B‐GFP Wnt reporter mouse. (B) Quantification of GFP fluorescence in AF cells (*n* = 3/sex/genotype). IIVD gene expression following *β‐catenin* deletion in aggrecan‐expressing cells in male and female mice. First row: Wnt signaling‐related genes include (C) *β‐catenin*, (D) *Dkk1*, (E) *Lef1*, and (F) *Ccnd1*. Second row: Inflammatory‐related genes include (G) *Ccl2*, (H) *Il1β*, (I) *S100a9*, (J) *Serpina1a*, and (K) *Il6*. Sample size: Male WT (*n* = 6–7), Male cKO^ACN^ (*n* = 5–6), Female WT (*n* = 5–6), Female cKO^ACN^ (*n* = 5–6). Data are presented as mean + SD. Gene expression was normalized to WT male IVDs. Male and female mice were compared by genotype with unpaired Student's *t*‐tests with astrerix denoting significance *p < 0.05, **p < 0.01, ***p < 0.001. Scale bar: 100 μm.

To further characterize *β‐catenin* deletion in aggrecan‐lineage cells, we determined the expression of genes related to Wnt signaling, pro‐inflammatory signaling, NP cell maturity, cell proliferation, and ECM metabolism. Consistent with the reduction in Wnt signaling indicated by decreased GFP expression, conditional deletion of *β‐catenin* in male IVD and female IVD downregulated *β‐catenin* gene expression by 65% and 90%, respectively (Figure 1C). Next, we confirmed the regulation of key targets of Wnt signaling. *Dkk1*, a negative regulator of the Wnt signaling pathway, is a direct target of the *β‐catenin*/TCF pathways, and *β‐catenin* deletion significantly increased *Dkk1* expression by 167% in male IVD while it was unaffected in female IVD (Figure 1D). Lef1 is a transcriptional mediator in the Wnt/*β‐catenin* signaling pathways, and deletion of *β‐catenin* significantly increased *lef1* gene expression by 294% and 172% in male and female IVDs, respectively (Figure 1E). *Ccnd1*, a proliferation marker regulated by Wnt signaling, was not affected by *β‐catenin* deletion (Figure 1F). These data indicated that tamoxifen dosing reduced IVD *β‐catenin* gene expression in this mouse model and suggested that there was compensatory upregulation of other Wnt signaling markers.

Dysregulation of Wnt/*β‐catenin* signaling is associated with chronic inflammation in many tissue systems (Jridi et al. 2020), but its role in the IVD was less clear. Here, *β‐catenin* deletion downregulated monocyte chemoattractant *ccl2* gene expression in male and female IVDs by 58% and 79%, respectively (Figure 1G). By contrast, *β‐catenin* deletion significantly upregulated pro‐inflammatory cytokine *il1b* by 385% and activated‐neutrophil marker *s100a9* by 200% in male IVDs, but deletion of *β‐catenin* did not affect their expression in female IVDs (Figure 1H,I). Anti‐inflammatory and neutrophil‐elastase inhibitor *serpinA1a*, and inflammatory cytokine *il6* were not regulated by *β‐catenin* deletion in male or female IVD (Figure 1J,K). Lastly, deletion of *β‐catenin* did not affect the gene expression for notochordal progenitor marker *tagln*, notochordal markers *T* (*brachyury*) and *krt8*, NP cell marker *cd109*, AF cell marker *scx* (Figure S4C–H), or any ECM related markers such as *acan, col1, col2, mmp3*, or *adamts5* (Figure S4I–M).

### 3.2 IVD‐Specific Deletion of *β‐Catenin* Reduced Myeloid Cell Presence in Naive IVD and Impaired Their Recruitment to Injured IVD

To track immune cell recruitment, mice were injected with a myeloid cell‐specific radiotracer and imaged by PET/CT at baseline and after IVD injury by TC. The PET/CT images showed uptake of the PERFECTA‐HDL tracer in baseline and post TC images, and the boxes highlighted CC7 and CC9, where the needles were implanted for TC (Figure 2A). The schematic identified the regions of interest quantified, where “Bone‐IVD‐Bone” included myeloid cells from portions of the upper and lower vertebrae and “IVD” highlighted myeloid cells only at the IVD of interest (Figure 2B). At baseline in male mice, *β‐catenin* deletion reduced signal intensity of both TC and control “Bone‐IVD‐Bone” region by 76% as well as TC and control IVD region by 79% and 76%, respectively (Figure 2C–E). In male mice, IVD injury (TC) increased signal intensity of the “Bone‐IVD‐Bone” and “IVD” regions of interest in WT mice by 1009% and 838%, respectively, and in cKO^ACN^ by 2209% and 2344%, respectively (Figure 2C,E). More importantly, deletion of *β‐catenin* attenuated IVD injury‐induced signal intensity in all measured regions and IVD by ~50%. The pattern of the response by deletion of *β‐catenin* and IVD injury was similar in female mice with the major exception that, while IVD injury by TC enhanced signal intensity in WT and cKO^ACN^ mice by 231% and 312%, respectively (Figure 2G,I), this response was larger in male mice (Figure 2G–J). The attenuated response to IVD injury in female mice dampened the impact of the absence of *β‐catenin* on IVD injury‐induced signal intensity to the site of injury, but deletion of *β‐catenin* impaired signal intensity to control “Bone‐IVD‐Bone” and “IVD” regions by 29% and 18%, respectively (Figure 2H,J).

**FIGURE 2:**
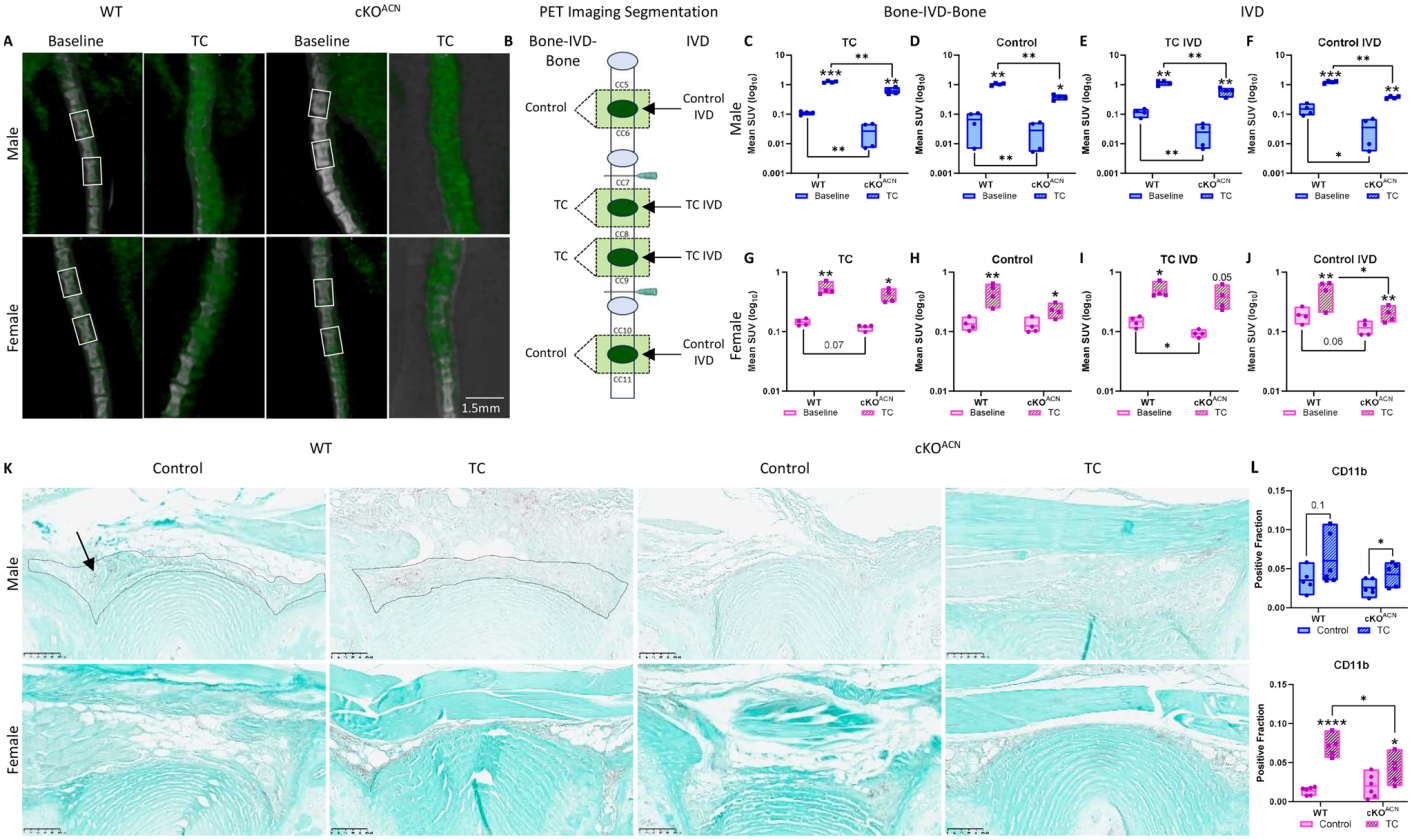
IVD‐specific deletion of *β‐catenin* reduced myeloid cell accumulation in naïve IVDs of male and female mice and reduced myeloid cell recruitment in response to IVD injury. (A) PET/CT images of (top row) male and (bottom row) female WT and cKO^ACN^ tails before and after tail compression (TC). Green indicated uptake of ^89^Zr‐PERFECTA‐HDL. White boxes highlighted the instrumented vertebrae CC7 and CC9. (B) The schematic of the tail indicated the placement of the pins and region of interest analysis, where ‘Bone‐IVD‐Bone’ segments were indicated by light green boxes and ‘IVD’ alone were indicated by dark green fill‐ins. Radiotracer standard uptake values (SUV) of (top row males, bottom row female) of the (C, G) injured (TC) and (D, H) adjacent control of the ‘Bone‐IVD‐Bone’ segments and of the (E, I) injured (TC) and (F, J) adjacent control of the ‘IVD’ segment of (C–F) male and (G–J) female mice. (K) Cd11b staining of male (top row) and female (bottom row), WT (left 2 panels) and *β‐catenin* cKO^ACN^ (right two panels) control and TC IVDs. (L) Quantification of cd11b staining in male (top graph) and female (bottom graph) AF + periphery with example ROI outlines and arrow pointing at positive staining. Statistics: Cross‐sectional data were compared separately using unpaired Student's *t*‐tests and longitudinal data were compared separately using paired *t*‐tests with asterisks denoting significance, *p < 0.05, **p < 0.01, ***p < 0.001, ****p < 0.0001. Data were presented as box plots, where the top and bottom line represent the first and third quartile with a line at the mean. Scale bar: (A) 1.5 mm, (K) 100 μm.

To corroborate PET imaging, WT and cKO^ACN^ IVD control and TC segments were stained for pan‐myeloid marker cd11b (Figure 2K). Although cd11b staining was not different between control WT and cKO^ACN^ IVD of male and female mice, TC injury increased cd11b staining in the periphery of the IVD of WT and cKO^ACN^ male mice by 65%–70% and female mice by 100%–400% (Figure 2L). Compared to the IVD injury response of WT, *β‐catenin* deletion attenuated the injury‐induced upregulation of cd11b staining by 30% in male mice and 42% in female mice. These data indicate that loss of IVD Wnt signaling reduced myeloid cell accumulation to naive and injured IVD.

### 3.3 NP‐Specific Deletion of *β‐Catenin* Upregulated Pathways Related to Developmental Biology and Immune Cells, and Downregulated Few Pathways

To determine if IVD injury‐induced impairment of immune signaling arises in NP cells—as it is believed that early degeneration starts in the NP (Gao et al. 2022)—we targeted deletion of *β‐catenin* in the NP using the SHHCreERT2 (Choi et al. 2008). The short‐term deletion of *β‐catenin* mildly regulated IVD transcriptomic signaling. Compared to WT control IVDs, deletion of *β‐catenin* in the NP significantly upregulated 55% of the top 20 pathways related to developmental biology, 25% related to metabolism, and 20% related to immune pathways (Figure S5A, Table S1). By contrast, deletion of *β‐catenin* downregulated pathways related to “Cellular response to external stimuli” (15%) with two significant pathways: “Response to purine‐containing compound” and “Response to organophosphorus”, and “Neuronal system” (40%) with 1 significant pathway: “Synaptic transmission, glutamatergic”, but none of the other 17 pathways were significant (Figure S5B, Table S2).

### 3.4 Deletion of *β‐Catenin* in NP Cells Altered the Transcriptomic Response to IVD Injury

The IVD transcriptome was subjected to Principal Component Analyses (PCA) to visualize similarities or dissimilarities of transcriptomes by IVD injury and genotype. Compared to the control IVD in WT mice, the clustering of the injured WT IVD was distinct, with no overlap (PC1 75%, PC2 12%; Figure 3A). The variation in control IVD could be due to baseline biological heterogeneity (Boubriak et al. 2013). Similarly, compared to the internal control IVD of cKO^SHH^ mice, the clustering of the injured IVD cKO^SHH^ mice was distinct, with no overlap (PC1 55%, PC2 24%; Figure 3B). Next, comparing control IVD transcriptomes, WT and cKO^SHH^ IVDs overlapped, indicating shared transcriptomic expression (PC1 64%, PC2 16%; Figure 3C). By contrast, when comparing all IVD in one PCA plot, there was no transcriptomic overlap between the injured WT IVD and the injured cKO^SHH^ IVD, indicating a *β‐catenin*‐dependent distinct response to IVD injury (PC1 17%, PC2 54%; Figure 3D).

**FIGURE 3:**
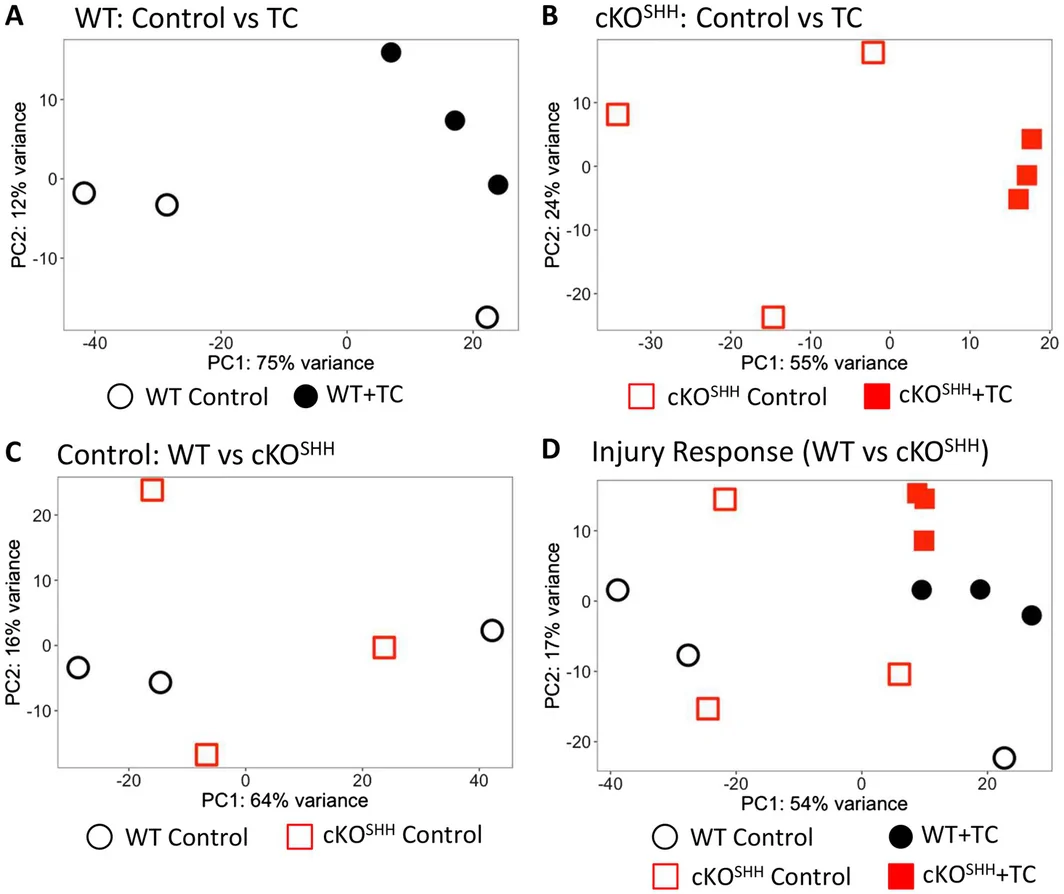
Deletion of *β‐catenin* in the nucleus pulposus distinctly regulated the transcriptome to IVD injury. PCA plots of (A) ‘Control vs. TC’ of WT IVDs, (B) ‘Control vs. TC’ of cKO^SHH^ IVDs, (C) ‘WT vs. cKO^SHH^’ of control IVDs, and (D) all IVDs with emphasis on TC of WT IVDs vs. TC of cKO^SHH^ IVDs. Each point on the graph represents the transcriptome of 2 IVDs from 1 mouse.

### 3.5 NP‐Specific Deletion of *β‐Catenin* Impaired IVD Injury‐Induced Upregulation of Immune Pathways

Using enrichGO, we examined which pathways were altered by IVD injury and by *β‐Catenin* deletion. Next, we analyzed the top 20 up‐ and down‐regulated pathways and searched the differentially expressed genes against the Reactome database to categorize each pathway. Heatmaps were chosen to reflect the main differentially regulated pathways in the top 20.

IVD Injury (TC) significantly upregulated 2967 pathways in WT IVDs and 2381 in cKO^SHH^ IVDs. Of these, 1992 pathways were shared, 975 were distinctly upregulated in WT IVDs, and 389 were distinctly upregulated in cKO^SHH^ IVDs (Figure 4A). In response to IVD injury, both WT and cKO^SHH^ mice shared similarities in upregulated pathways. For instance, among the top 20 upregulated pathways, ‘Cell Cycle’ accounted for 25% in WT IVDs and 80% in cKO^SHH^. Similarly, 15% of pathways in WT IVDs and 10% of pathways in cKO^SHH^ IVDs were related to ‘Signaling Transduction’ (Table S1). Further, IVD injury similarly upregulated “Regulation of Wound Healing” (FE = 3.6 for WT and FE = 3.6 for cKO^SHH^, FDR < 0.0001, Figure S6A).

**FIGURE 4:**
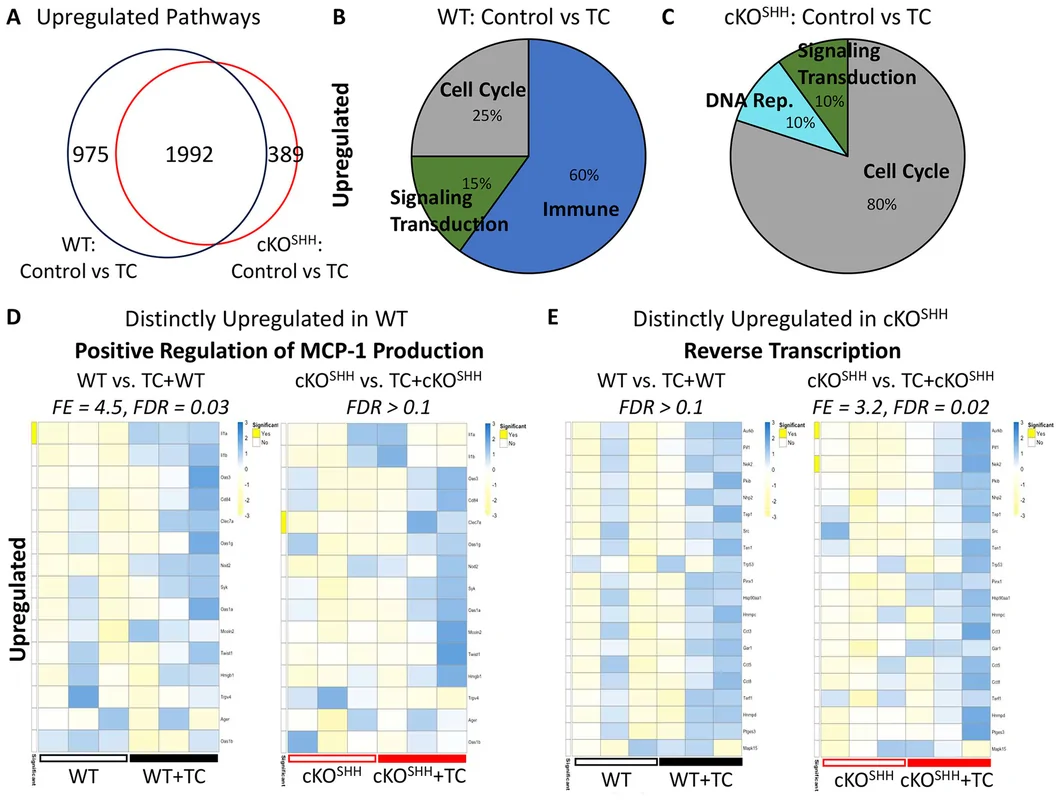
IVD injury differentially upregulated pathways in WT and cKO^SHH^ IVDs. (A) The number of pathways upregulated by tail compression (TC) in WT and cKO^SHH^ IVDs and categorized by the top 20 significantly upregulated pathways by injury in (B) WT mice and (C) cKO^SHH^ mice. Heat maps of (D) ‘Positive Regulation of MCP‐1 Production’ and (E) ‘Reverse Transcription.’ Yellow indicated low gene expression and blue indicated high gene expression. DNA Rep., DNA replication.

By contrast, 60% of the top 20 pathways in WT IVDs upregulated by IVD injury were immune‐related, whereas these pathways were absent in cKO^SHH^ IVDs (Figure 4B,C, Table S3). More specifically, injury in WT IVDs upregulated “Positive Regulation of Monocyte Chemotactic Protein‐1 Production” (FE = 4.5, FDR = 0.03), but IVD injury did not significantly upregulate this pathway in cKO^SHH^ IVDs (Figure 4D). Monocyte chemotactic protein‐1 (MCP‐1) is a chemokine that regulates and promotes the migration and infiltration of monocytes/macrophages to sites of inflammation (Deshmane et al. 2009). Genes distinctly regulated by IVD injury in the WT group included *saa3, bmp2*, and *cxcl2* related to neutrophil chemotaxis (Deshmane et al. 2009) and *ptges* involved in inflammation and pain (Table S4). Conversely, IVD injury distinctly upregulated “Reverse Transcription” (FE = 3.2, FDR = 0.02) in cKO^SHH^ IVD with related genes like *pole* and *twist2* being upregulated (Figure 4E, Table S4).

### 3.6 NP‐Specific Deletion of *β‐Catenin* Promoted Enhanced Downregulation of Signaling Transduction Pathways in Response to IVD Injury

IVD injury significantly downregulated a total of 860 pathways in WT IVDs and 895 in cKO^SHH^ IVDs. 446 of these downregulated pathways were shared between WT and cKO^SHH^ IVDs, 414 pathways were distinctly downregulated in WT IVDs and 449 of these were distinctly downregulated in cKO^SHH^ IVDs (Figure 5A). Extracellular matrix‐related pathways constituted 20% of the top 20 downregulated pathways by IVD injury in WT and cKO^SHH^ IVDs (Figure 5B,C). For instance, IVD injury (FDR < 0.001) impaired “Extracellular Matrix Organization” (FE = 4.2 for WT and FE = 4.2 for cKO^SHH^), significantly downregulating key ECM‐related genes, such as *grem1* and *ecm2* (Figure S6B). By contrast, compared to injured IVD of WT mice, deletion of *β‐Catenin* exacerbated the number of ‘Signal Transduction’ pathways downregulated by IVD injury (20% vs. 70%) (Figure 5B,C).

**FIGURE 5:**
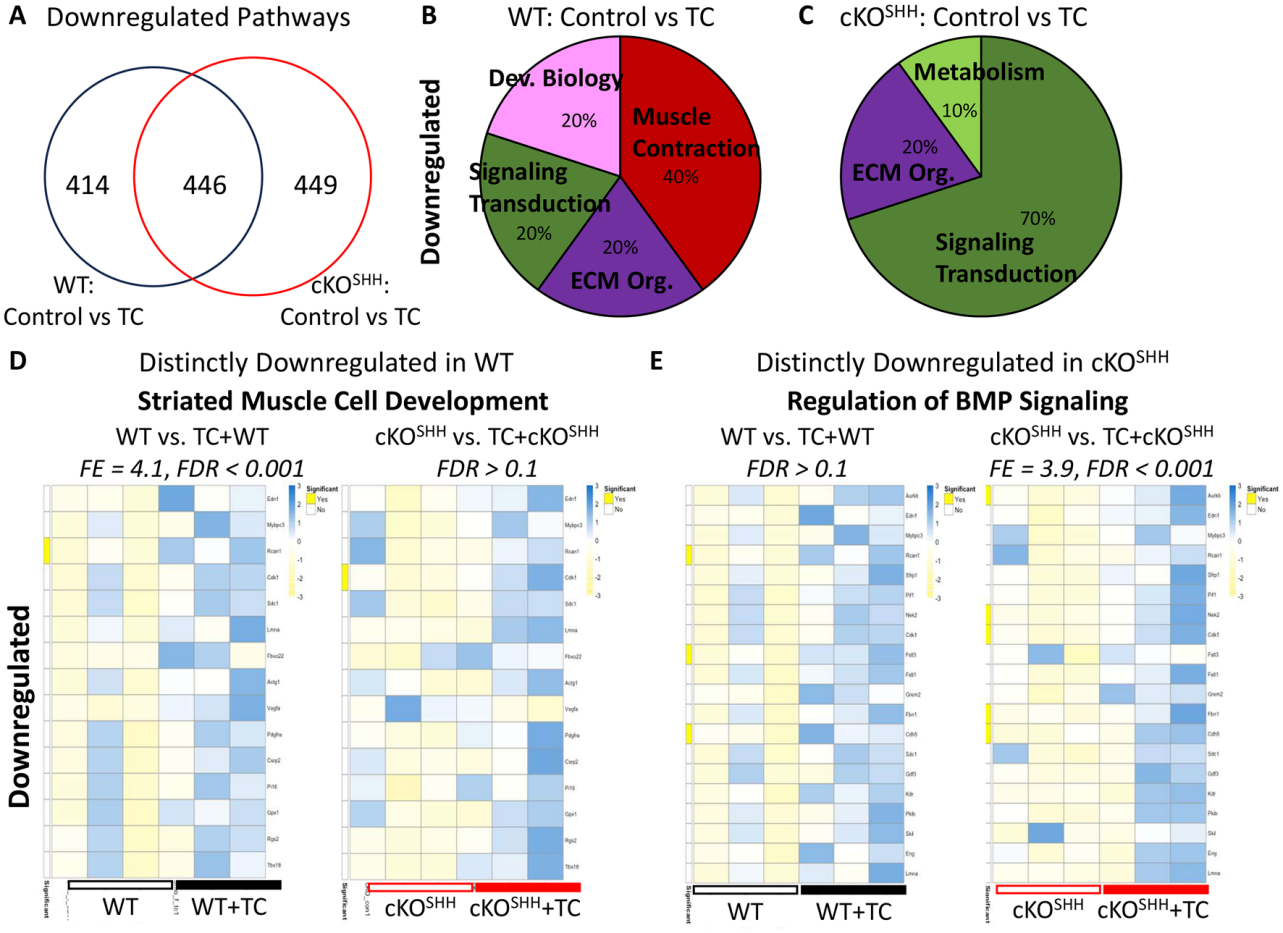
IVD injury differentially downregulated pathways in WT and cKO^SHH^ IVDs. (A) The number of pathways downregulated by tail compression (TC) in WT and cKO^SHH^ IVDs and categorized by the top 20 significantly downregulated pathways by injury in (B) WT mice and (C) cKO^SHH^ mice. Heat maps of (D) ‘Striated Muscle Cell Development’ and (E) ‘Regulation of BMP Signaling.’ Yellow indicated low gene expression and blue indicated high gene expression. Dev Biology, developmental biology; ECM Org, ECM organization.

In WT IVDs, of the top 20 pathways downregulated by IVD injury, 20% were related to ‘Developmental Biology’, and the largest number (40%) were related to ‘Muscle Contraction’. One such pathway was “Striated Muscle Cell Development” (FE = 4.1, FDR < 0.001) (Figure 5D). Here, some of the top significantly downregulated genes were *ablim3, slc24a2*, and *cacna1a*, which may be related to cell and actomyosin contractilit*y* (Pardo‐Pastor et al. 2018) (Table S5). In cKO^SHH^ IVDs, ‘Signaling Transduction’ constituted (70%) the greatest number of pathways downregulated by IVD injury. Here, IVD injury distinctly significantly downregulated “Regulation of BMP Signaling” (FE = 3.9, FDR < 0.0001) (Figure 5E), with uniquely downregulated genes such as *wsip3, chadl*, and *hhip* (Table S5).

### 3.7 NP‐Specific Deletion of *β‐Catenin* Impaired Pro‐Inflammatory Transcription and Downregulated the Transcription of Chemotactic Inhibitor *SerpinA1a* in Response to IVD Injury

Based on an FDR < 0.1 and |log_2_FC| ≥ 1, we analyzed the transcriptomes of WT and cKO^SHH^ IVD subjected to TC. IVD injury upregulated 109 shared genes between WT and cKO^SHH^ mice, while distinctly upregulating 220 genes in WT mice and 178 genes in cKO^SHH^ mice (Figure 6A). IVD injury initiates wound healing, and both groups showed high upregulation of genes related to IVD “ECM Remodeling”, such as *adamts5, adamts7* and *mmp3*. Some of the highest unique differentially regulated genes by injury in WT IVDs include *saa3, nos2*, and *il1β* indicating a strong immune response and these genes were not upregulated by IVD injury in cKO^SHH^ IVDs (Figure 6C, Table S4). Conversely, in cKO^SHH^ IVD, injury distinctly upregulated genes related to cell proliferation and differentiation such as *myog, twist2*, and *pole* (Figure 6D, Table S4). IVD injury downregulated 168 genes in common between WT and cKO^SHH^ IVD, distinctly downregulating 140 genes in WT IVD and 369 genes in cKO^SHH^ IVD (Figure 6B). IVD injury uniquely downregulated genes related to signaling transduction in WT IVD, such as *foxf1* and *svopl* (Figure 6C) and, in cKO^SHH^ IVD, such as *fam101a, wisp3*, and *hhip* (Table S5). Together, these data indicate that deletion of *β‐catenin* altered the expression of genes related to immune cell recruitment and tissue homeostasis in response to IVD injury.

**FIGURE 6:**
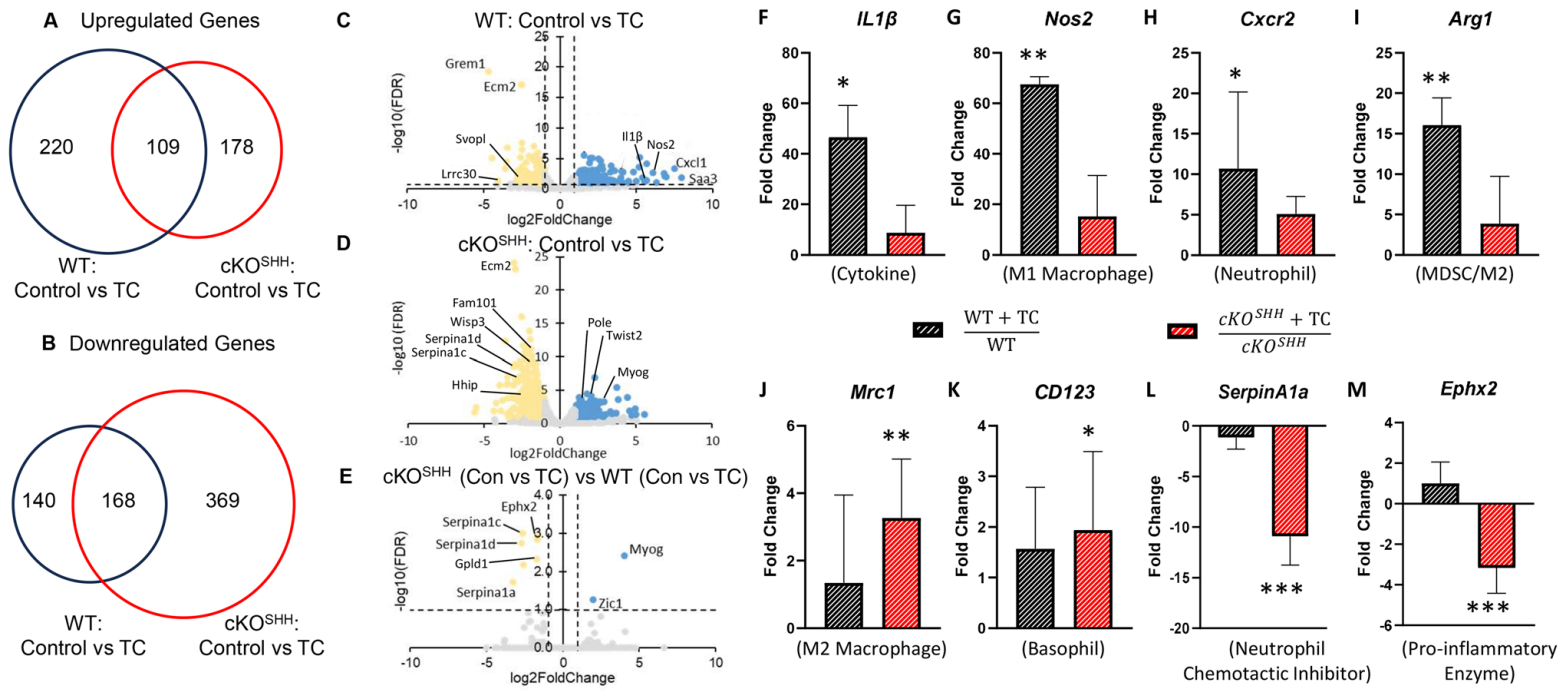
(A) Genes significantly upregulated and (B) downregulated by IVD injury in WT and cKO^SHH^ IVDs. Volcano plots of genes significantly that are regulated by IVD injury (C) in WT IVDs, (D) in cKO^SHH^ IVDs, and (E) comparing the response to IVD injury of cKO^SHH^ IVDs relative to WT IVDs. Plots of immune cell‐related markers in response to IVD injury of WT IVD and cKO^SHH^ IVD for (F) pro‐inflammatory cytokine marker *Il1β*, (F) M1 macrophage marker *nos2*, (H) neutrophil chemokine receptor *cxcr2*, (I) myeloid‐derived suppressor cells (MDSC)/M2 macrophage marker *arg1*, (J) M2 macrophage marker *mrc1*, (K) basophil marker *cd123*, (L) anti‐inflammatory neutrophil inhibitor *SerpinA1a*, and (M) pro‐inflammatory enzyme *ephx2*. Data are presented as mean ± SD and normalized to WT. *FDR < 0.05, **FDR < 0.01, ***FDR < 0.000001.

Strikingly, when directly comparing the relative IVD injury response between WT and cKO^SHH^ IVDs, five of the seven top downregulated genes were related to inflammation: serpinA1 genes, *ephx2*, and *gpld1* (Figure 6E). In the mouse, there are five isoforms of this gene *serpinA1a* through *serpinA1e*. Compared to the lack of regulation of *serpinA1* genes by IVD injury in WT IVD, deletion of *β‐catenin* with IVD injury significantly downregulated *serpinA1a*, *1c*, and *1d*. Alpha‐1 antitrypsin protein, which is encoded by *SERPINA1*, inhibits neutrophil‐derived elastase and limits ECM degradation (Hazari et al. 2017). Similarly, compared to the lack of regulation of *ephx2* by IVD injury in WT IVD, deletion of *β‐catenin* with IVD injury downregulated *ephx2. Ephx2* breaks down epoxyeicosatrienoic acids (EETs), converting EETs from anti‐inflammatory to pro‐inflammatory diols. Finally, *β‐catenin* deletion with IVD injury downregulated *gpld1. Gpld1* is a liver‐derived enzyme that is linked to counteracting age‐related inflammation by cleaving glycosylphosphatidylinositol (GPI)‐anchored protein (Wen et al. 2025). Both *serpinA1* genes and *gpld1* have been identified in proteomic studies of the IVD (Tam et al. 2020).

### 3.8 Deletion of *β‐Catenin* Shifted the Transcriptomic Response to IVD Injury From Early Response to Late Response Immune Cells

To determine the potential immune cell types recruited by IVD injury, we plotted key genes from the bulk sequencing of WT and *β‐catenin*‐deficient IVDs in response to IVD injury. Injury of WT IVD upregulated cytokine marker *il1*β by nearly 40‐fold, M1 macrophage marker *nos2 by* over 50‐fold, neutrophil chemotactic factor and receptor pair *cxcl2* and *cxcr2* by almost 100‐ and 5.6‐fold, respectively, and *arg1* by 12‐fold (Figure 6F–I). The upregulation of neutrophil‐related markers may indicate that *arg1* reflected myeloid‐derived suppressor cells rather than anti‐inflammatory M1 macrophages. Conversely, IVD injury of *β‐catenin*‐deficient IVDs upregulated M2 macrophage marker *mrc1 by* twofold, basophil marker *cd123* by 4.3‐fold, neutrophil chemotactic inhibitor *serpinA1a* by 10‐fold, and *ephx2* by 4.2‐fold (Figure 6J–M).

### 3.9 Aging Impaired Cytokine Expression and Altered Chemotactic Gene Expression in Response to IVD Injury

Lastly, to corroborate that loss of IVD Wnt signaling models the aged impairment of chemotactic signaling with IVD injury, 5‐, 12‐, and 24‐month‐old female and male C57BL/6 mice were subjected to TC. First, IVD injury upregulated pro‐inflammatory cytokine marker *il6* across ages in both female and male IVD (Figure S7A–A″, top row). Similar to *β‐catenin*‐deficient IVDs, aging attenuated the IVD injury‐induced upregulation of *il6* in female mice. Further, the IVD injury‐induced regulation of chemotactic factor *serpinA1a* was differentially affected by age. Young‐adult IVD did not regulate *serpinA1a* in response to IVD injury, middle‐aged mice upregulated *serpinA1a* in response to injury, and old female mice downregulated *serpinA1a* while aged male mice upregulated *serpinA1a* (Figure S7A–A″, bottom row). Similar to *β‐catenin*‐deficient IVD, aging altered the gene expression of cytokines and chemokines in responses to IVD injury.

## 4 Discussion

We examined the in vivo transcriptomic response and myeloid cell recruitment in inflammatory IVDD between young‐adult IVD and young‐adult *β‐catenin*‐deficient IVDs as a model of Wnt signaling‐deficient aged IVD cells without the systemic inflammatory milieu of aging. IVD injury promotes neo‐vascularization and de novo innervation (Walk et al. 2024) by pro‐inflammatory cytokine expression of IL‐1β, IL‐6, TNF‐α and nuclear factor‐kappa B (NF‐Κβ) signaling to also recruit a cascade of infiltrating neutrophils, monocytes and other myeloid‐derived cells to begin the healing process (Clayton, Walk, et al. 2025). Similarly, IVD injury upregulated cytokine expression, activated leukocyte migration pathways, and promoted myeloid cell recruitment of young‐adult IVD. Whereas, deletion of *β‐catenin* attenuated pro‐inflammatory chemotactic transcription, accentuated pathways related to cell differentiation and proliferation, and reduced myeloid‐derived cell recruitment upon IVD injury.

These data suggest that painful age‐related IVDD (Fujii et al. 2019) may occur through Wnt signaling‐deficient aged IVD cells by attenuating excessive neutrophil recruitment during natural ‘inflammaging,’ which is characterized by elevated circulating cytokines and monocytes (Reitsema et al. 2023). In the aged, high levels of circulating IL6 and TNFα in serum are associated with an increased risk of IVDD (Bruunsgaard 2002). In vivo deletion of Wnt signaling transcription factor *β‐catenin* in SHH‐expressing NP cells modeled the Wnt signaling deficiency of aged human NP cells, the age‐related functional IVD degradation, and the impaired pro‐inflammatory signaling from in vitro NP cell suppression of Wnt signaling (Hiyama et al. 2013). Therefore, attenuated immune cell recruitment by Wnt signaling‐deficient aged IVD cells may attenuate excessive discogenic, painful immune cell recruitment (Parisien et al. 2022), but prolonged anti‐inflammatory signaling may impair appropriate immune cell recruitment and proper IVD healing (Clayton, Sebastian, et al. 2025). Further, mature NP cells have a lower capacity to produce and maintain the ECM (Liang et al. 2022); therefore, aged IVD ECM remodeling may be driven by IVD cell‐intrinsic changes, that alter inflammatory signaling and myeloid cell infiltration. These data may account for the highly variable age‐related pain behavior observed in mice (Millecamps et al. 2020) and incidence of cLBP with variable grades of IVDD (Boden et al. 1990; Fujii et al. 2019).

By sex, deletion of *β‐catenin* impaired myeloid‐derived cell intensity in the injured IVD of male mice more so than in the injured IVD of female mice. Similarly, the IVD gene expression for myeloid marker *Cd11b* and IVD‐infiltrating neutrophils in response to IVD injury was less in female mice than in male mice. While the elevated incidence of cLBP in older women compared to men may be associated with menopause (Wang 2017), our data and the literature (Clayton, Walk, et al. 2025) suggest that the greater incidence of cLBP in young‐adult men than young‐adult women may be associated with greater immune cell accumulation.

NP‐specific deletion of *β‐catenin* during IVD injury potently reduced the transcriptomic expression of (i) *ephx2* and (ii) *serpinA1* paralogs, both of which have emerging anti‐inflammatory roles in immune cell recruitment and IVDD. First, epoxyeicosatrienoic acids (EETs) are metabolites of arachidonic acid, which are a target of nonsteroidal anti‐inflammatory drugs that block nociception, inhibit NF‐Κβ signaling, and minimize inflammatory IVDs (Li et al. 2017). EETS are degraded by soluble epoxide hydrolase, which is encoded by *EPHX2*. Multi‐omics approaches suggest that elevated arachidonic acid activity promotes intercellular communication with NP cells contributing to inflammation and disease progression (Tan et al. 2023). Alpha‐1 antitrypsin (AAT) is an acute phase responder that inhibits myeloid cell chemotaxis (Guttman et al. 2015) and modulates several pro‐inflammatory cytokines, whereas, defective AAT impairs IL‐6 production. In IVD injury, *β‐catenin*‐deficient IVD cells downregulated *Ephx2*, may have suppressed inflammatory signaling and reduced neutrophil recruitment. Further, *β‐catenin*‐deficient IVD cells in response to IVD injury suppressed *SerpinA1* genes, which may have increased neutrophil elastase activity resulting in ECM degradation, promoting anti‐inflammatory macrophages, and impaired inflammatory resolution (Belchamber et al. 2020).

Wnt signaling is a potent regulator of cell differentiation and proliferation (Liu et al. 2022), and its absence in IVD injury promoted the upregulation of transcription factors that promote the differentiation of non‐IVD skeletal stem cells. IVD injury in wildtype mice upregulated pathways related to the cell cycle, but deletion of the Wnt signaling transcription factor *β‐catenin* further increased the number of “Cell Cycle”‐related pathways in the top 20. Corroboratively, IVD injury in wildtype mice downregulated *zic1*, whereas deletion of *β‐catenin* in IVD injury upregulated *zic1*. *Zic1* is a recently discovered transcription factor expressed in spinal‐specific skeletal stem cells, including the vertebra and AF (Sun et al. 2023), whose overexpression promotes osteogenic and adipogenic commitment (Thottappillil et al. 2023). Similarly, the downregulation of muscle‐related pathways in wildtype IVD in response to injury was attenuated in conditional KO IVD, as corroborated by strong upregulation of *myod1* and *myog*, master regulators of muscle stem cell differentiation.

This preclinical study has some inherent limitations based on the models and approaches. First, we deleted *β‐catenin* in young‐adult IVD to model age‐related IVD loss of Wnt signaling and corroborated that aged IVD similarly impair injury‐induced pro‐inflammatory signaling, but aged IVD injury was conducted for 1 day rather than 1 week as conducted in the KO models to determine if the age‐related attenuated response occurred early. Nevertheless, future studies will need to determine IVD injury‐induced IVDD in Wnt signaling‐deficient IVD. Next, both male and female mice incur age‐related IVDD and Wnt signaling‐deficiency (Hutchinson et al. 2023), but low sample size may have impaired confirmation of loss of Wnt signaling in the IVD by deletion of *β‐catenin*. We confirmed that deletion of *β‐catenin* reduced its gene expression in male and female IVD, but loss of Wnt signaling by GFP expression was noted in male AF cells, and not in female AF cells. We included sex as a biological variable in most of our outcomes, except for the bulk sequencing, but the general effect of deletion of *β‐catenin* on chemotactic signaling and myeloid recruitment in response to IVD injury was similar between sexes. Next, bulk sequencing of whole IVDs does not transcriptomically differentiate cellular identity, but we confirmed NP‐specific deletion of *β‐catenin*, and previous data confirm the genetic efficiency of the deletion (Holguin and Silva 2018). Next, although the transcriptomic IVD injury‐induced response to *β‐catenindeficiency* using the Shh‐creERT2 may not fully represent the signaling from full age‐related loss of IVD cell Wnt signaling (Silva and Holguin 2020), the data suggest (i) that early age‐related loss of Wnt signaling in the NP (Dahia et al. 2009) may impair inflammatory signaling and (ii), NP‐specific loss of *β‐catenin* would also impair myeloid‐derived cell recruitment as both NP‐ and IVD‐specific deletion of *β‐catenin* reduced MCP1/*ccl2* transcriptomic expression.

Overall, we identified a potential cellular mechanism by which aged Wnt signaling‐deficient IVDs may promote IVDD and cLBP. We previously found that deletion of Wnt signaling transcription factor *β‐catenin* in IVD cells weakens IVD function, which may promote IVDD (Holguin and Silva 2018) and, here, we show that it may also mitigate pro‐inflammatory gene expression and attenuate immune cell recruitment in response to IVD injury. These data suggest that effective treatment strategies for age‐related discogenic cLBP would benefit from understanding the balance between systemic inflammation and IVD chemotactic signaling for immune cell recruitment.

## Author Contributions

Conceptualization: T.M.K., N.H. Study design: T.M.K., N.H. Resource acquisition: N.H. Performed experiments: T.M.K., J.M., A.R., M.U., W.W. Analyzed data and prepared figures: T.M.K., J.J.H., J.M. Interpreted results and contributed to figure edits: T.M.K., A.J.P.T., N.H. Funding acquisition: N.H. Project administration: T.M.K., N.H. Supervision: T.M.K., N.H. Writing – original draft: T.M.K., J.J.H., A.J.P.T., N.H. Writing – review, editing and approval: T.M.K., A.J.P.T., N.H.

## Funding

This work was funded by NIH/NIAMS grants: R01 AR078764 (N.H.).

## Conflicts of Interest

The authors declare no conflicts of interest.

## Supporting information


**Figure S1:** Postoperative X‐ray image checking the instrumentation of needle placement for the tail compression (TC) device. 23‐gauge needles transcutaneously placed perpendicular through CC7 and CC9. IVDs used for bulk RNASequencing—Red IVDs (between CC7‐8 and CC8‐9) indicate tail compressed (TC) samples and blue IVD (between CC5‐6 and CC10–11) indicate internal control.

**Figure S2:** Schematic of dissection. Above shows how the tail was dissected for histology. Cutting through the IVD between CC4‐5 and CC6‐7 and cutting through the IVD between CC9‐10 and CC11–12, these segments were used as internal controls. The motion segment that remains between CC7‐CC9 serves as the tail compressed (TC) sample.

**Figure S3:** Transverse slice from PET/CT scans. (A) Blue outlining the vertebral body (bright white) representing the bone portion contoured for Bone‐IVD‐Bone segments. (B) Yellow outlining the IVD (lack of bright white) representing the IVD portion contoured. Scale bar 1 mm.

**Figure S4:** (A) Images of GFP expression merged with Dapi from the TCF/Lef:H2B‐GFP Wnt reporter mouse. Sample size: *n* = 3/sex/genotype. IVD gene expression following *β‐catenin* deletion in aggrecan‐lineage cells in male and female mice. First row: IVD cell type markers include (B) Tagln, (C) T (brachyury), (D) Krt8, (E) Cd9, (F) Cd109, and (G) Scx. Second row: Extracellular matrix related genes include (H) Acan, (I) Col1, (J) Col2, (K) Mmp3, and (L) Adamts5. Sample size: Male WT (*n* = 6–7), Male cKO^ACN^ (*n* = 5–6), Female WT (*n* = 5–6), Female cKO^ACN^ (*n* = 5–6). Data are presented as mean + SD. Gene expression was normalized to WT male IVDs. Male and female mice were compared by genotype with unpaired student *t*‐tests. Trending statistical comparisons of *p* < 0.01 were included and significance was noted below *p* < 0.05. Scale bar: 100 μm.

**Figure S5:** Baseline comparisons of WT and cKO^SHH^ IVDs without injury of (A) pathways upregulated and (B) downregulated by *β‐catenin* deletion in NP cells. Dev. Biology = Developmental Biology. Trans. Of Small Molec. = Transport of Small Molecules. Vesc. Med. Trans., vesicle mediated transport; Cell. Resp. to Ext. Stim., cellular response to external stimuli.

**Figure S6:** Injury‐induced shared (A) upregulated pathways and (B) downregulated pathways in WT and cKO^SHH^ IVDs. Yellow indicates low gene expression and blue indicates high gene expression.

**Figure S7:** Top Row—IL6 gene expression and bottom row—SerpinA1a gene expression in (A) WT and cKO^SHH^ subjected to 1 week TC, (A') female 5‐, 12‐, and 24‐month‐old WT Control and 24‐h TC IVDs, and (A″) male 5‐, 12‐, and 24‐month‐old WT Control and 24‐h TC IVDs. In (A) *FDR < 0.05, **FDR < 0.01, ***FDR < 0.000001. In (A', A″) *p < 0.05, **p < 0.01.

**Table S1:** Top 20 injury‐induced and control upregulated GO terms.

**Table S2:** Top 20 injury‐induced and control downregulated GO terms.

**Table S3:** Ranks of Immune Pathways Regulated by Injury in WT and cKO^SHH^ IVD.

**Table S4:** Top 40 uniquely injury‐induced upregulated genes.

**Table S5:** Top 40 uniquely injury‐induced downregulated genes.

## Data Availability

The non‐sequencing data are available in the main text and the Supporting Information. The bulk RNA‐seq dataset generated in this study is publicly available at Gene Expression Omnibus.
