# Supplementary material for: β‐Catenin‐Deficient Intervertebral Disc Cells Reduce Chemotactic Transcription and Myeloid Cell Recruitment to Injured Discs of Mice
Source: Aging Cell. 2026 Oct 1;25(10):e70740. doi: 10.1111/acel.70740 (PMC13631559; doi:10.1111/acel.70740)
Supplement: Supplementary file 1 [file ACEL-25-e70740-s001.docx]

***Supplemental Figure 4.*** *(A) Images of GFP expression merged with Dapi from the TCF/Lef:H2B-GFP Wnt reporter mouse. Sample size: n=3/sex/genotype. IVD gene expression following β-catenin deletion in aggrecan-lineage cells in male and female mice. First row: IVD cell type markers include (B) Tagln, (C) T (brachyury), (D) Krt8, (E) Cd9, (F) Cd109, and (G) Scx. Second row: Extracellular matrix related genes include (H) Acan, (I) Col1, (J) Col2, (K) Mmp3, and (L) Adamts5. Sample size: Male WT (n=6-7), Male cKO^ACN^ (n=5-6), Female WT (n=5-6), Female cKO^ACN^ (n=5-6). Data are presented as mean + SD. Gene expression was normalized to WT male IVDs. Male and female mice were compared by genotype with unpaired student t-tests. Trending statistical comparisons of p<0.01 were included and significance was noted below p<0.05. Scale bar: 100 µm.*
