# Supplementary material for: β‐Catenin‐Deficient Intervertebral Disc Cells Reduce Chemotactic Transcription and Myeloid Cell Recruitment to Injured Discs of Mice
Source: Aging Cell. 2026 Oct 1;25(10):e70740. doi: 10.1111/acel.70740 (PMC13631559; doi:10.1111/acel.70740)
Supplement: Supplementary file 3 [file ACEL-25-e70740-s003.docx]

***Supplemental Figure 6.*** *Injury-induced shared (A) upregulated pathways and (B) downregulated pathways in WT and cKO^SHH^ IVDs. Yellow indicates low gene expression and blue indicates high gene expression.*
