# Supplementary material for: β‐Catenin‐Deficient Intervertebral Disc Cells Reduce Chemotactic Transcription and Myeloid Cell Recruitment to Injured Discs of Mice
Source: Aging Cell. 2026 Oct 1;25(10):e70740. doi: 10.1111/acel.70740 (PMC13631559; doi:10.1111/acel.70740)
Supplement: Supplementary file 4 [file ACEL-25-e70740-s004.docx]

***Supplemental Figure 2 -*** *Schematic of dissection. Above shows how the tail was dissected for histology. Cutting through the IVD between CC4-5 and CC6-7 and cutting through the IVD between CC9-10 and CC11-12, these segments were used as internal controls. The motion segment that remains between CC7-CC9 serves as the tail compressed (TC) sample.*
