# Supplementary material for: β‐Catenin‐Deficient Intervertebral Disc Cells Reduce Chemotactic Transcription and Myeloid Cell Recruitment to Injured Discs of Mice
Source: Aging Cell. 2026 Oct 1;25(10):e70740. doi: 10.1111/acel.70740 (PMC13631559; doi:10.1111/acel.70740)
Supplement: Supplementary file 5 [file ACEL-25-e70740-s005.docx]

***Supplemental Figure 3.*** *Transverse slice from PET/CT scans. A) Blue outlining the vertebral body (bright white) representing the bone portion contoured for Bone-IVD-Bone segments. B) Yellow outlining the IVD (lack of bright white) representing the IVD portion contoured. Scale bar 1 mm*
