# Supplementary material for: β‐Catenin‐Deficient Intervertebral Disc Cells Reduce Chemotactic Transcription and Myeloid Cell Recruitment to Injured Discs of Mice
Source: Aging Cell. 2026 Oct 1;25(10):e70740. doi: 10.1111/acel.70740 (PMC13631559; doi:10.1111/acel.70740)
Supplement: Supplementary file 6 [file ACEL-25-e70740-s006.docx]

***Supplemental Figure 7.*** *Top Row – IL6 gene expression and bottom row – SerpinA1a gene expression in (A) WT and cKO^SHH^ subjected to 1 week TC, (A’) female 5-, 12-, and 24-month-old WT Control and 24-hour TC IVDs, and (A’’) male 5-, 12-, and 24-month-old WT Control and 24-hour TC IVDs. In (A) *FDR<0.05, **FDR<0.01, ***FDR<0.000001. In (A’, A’’) *p<0.05, **p<0.01.*
