# Supplementary material for: β‐Catenin‐Deficient Intervertebral Disc Cells Reduce Chemotactic Transcription and Myeloid Cell Recruitment to Injured Discs of Mice
Source: Aging Cell. 2026 Oct 1;25(10):e70740. doi: 10.1111/acel.70740 (PMC13631559; doi:10.1111/acel.70740)
Supplement: Supplementary file 7 [file ACEL-25-e70740-s007.docx]

Top 20 upregulated pathways in WT: Control vs TC, cKO^SHH^: Control vs TC, and Control: WT vs cKO^SHH^. Categorized and sorted by p-adj.

Table S1. Top 20 injury-induced and control upregulated GO terms
