# Supplementary material for: β‐Catenin‐Deficient Intervertebral Disc Cells Reduce Chemotactic Transcription and Myeloid Cell Recruitment to Injured Discs of Mice
Source: Aging Cell. 2026 Oct 1;25(10):e70740. doi: 10.1111/acel.70740 (PMC13631559; doi:10.1111/acel.70740)
Supplement: Supplementary file 8 [file ACEL-25-e70740-s008.docx]

***Supplemental Figure 1.*** *Post-operative X-ray image checking the instrumentation of needle placement for the tail compression (TC) device. 23-gauge needles transcutaneously placed perpendicular through CC7 and CC9. IVDs used for bulk RNASequencing - Red IVDs (between CC7-8 and CC8-9) indicate tail compressed (TC) samples and blue IVD (between CC5-6 and CC10-11) indicate internal control samples.*
