# Supplementary material for: β‐Catenin‐Deficient Intervertebral Disc Cells Reduce Chemotactic Transcription and Myeloid Cell Recruitment to Injured Discs of Mice
Source: Aging Cell. 2026 Oct 1;25(10):e70740. doi: 10.1111/acel.70740 (PMC13631559; doi:10.1111/acel.70740)
Supplement: Supplementary file 9 [file ACEL-25-e70740-s009.docx]

Top 40 uniquely downregulated genes in response to injury in WT and cKO^SHH^ IVDs. Ranked by FDR.

Table S5. Top 40 uniquely injury-induced downregulated genes
