# Supplementary material for: β‐Catenin‐Deficient Intervertebral Disc Cells Reduce Chemotactic Transcription and Myeloid Cell Recruitment to Injured Discs of Mice
Source: Aging Cell. 2026 Oct 1;25(10):e70740. doi: 10.1111/acel.70740 (PMC13631559; doi:10.1111/acel.70740)
Supplement: Supplementary file 10 [file ACEL-25-e70740-s010.docx]

Table S4. Top 40 uniquely injury-induced upregulated genes

Top 40 uniquely upregulated genes in response to injury in WT and cKO^SHH^ IVDs. Ranked by FDR.
