# Supplementary material for: β‐Catenin‐Deficient Intervertebral Disc Cells Reduce Chemotactic Transcription and Myeloid Cell Recruitment to Injured Discs of Mice
Source: Aging Cell. 2026 Oct 1;25(10):e70740. doi: 10.1111/acel.70740 (PMC13631559; doi:10.1111/acel.70740)
Supplement: Supplementary file 11 [file ACEL-25-e70740-s011.docx]

Immune related pathways that reside in the top 20 pathways regulated by injury in WT IVD ranked by p-adj compared to the rank of the same pathway regulated by injury in cKO^SHH^ IVD.

Table S3. Ranks of Immune Pathways Regulated by Injury in WT and cKO^SHH^ IVD
