# Supplementary material for: β‐Catenin‐Deficient Intervertebral Disc Cells Reduce Chemotactic Transcription and Myeloid Cell Recruitment to Injured Discs of Mice
Source: Aging Cell. 2026 Oct 1;25(10):e70740. doi: 10.1111/acel.70740 (PMC13631559; doi:10.1111/acel.70740)
Supplement: Supplementary file 12 [file ACEL-25-e70740-s012.docx]

***Supplemental Figure 5.*** *Baseline comparisons of WT and cKO^SHH^ IVDs without injury of (A) pathways upregulated and (B) downregulated by β-catenin deletion in NP cells. Dev. Biology = Developmental Biology. Trans. Of Small Molec. = Transport of Small Molecules. Vesc. Med. Trans. = Vesicle Mediated Transport. Cell. Resp. to Ext. Stim. = Cellular Response to External Stimuli.*
